# How does patient-centered hospital culture affect clinical physicians’ medical professional attitudes and behaviours in Chinese public hospitals: a cross-sectional study?

**DOI:** 10.1186/s12910-023-00936-7

**Published:** 2023-08-02

**Authors:** Jing Chen, Qiu-xia Yang, Rui Zhang, Yan Tan, Yu-chen Long

**Affiliations:** 1 School of Medical and Health Management, Tongji Medical College, Huazhong University of Science & Technology, #13 Hangkong Road, Wuhan, Hubei Province 430030 P. R. China; 2 Research Center for Hospital High-Quality Development, Key Disciplinary Platform for the Implementation of Double First-Class Initiative in Liberal Arts of Huazhong University of Science and Technology, #13 Hangkong Road, Wuhan, Hubei Province 430030 P. R. China

**Keywords:** Medical Professionalism, Professional Attitude, Professional Behaviour, Organizational Culture, Hospital Culture, Patient-centered, China

## Abstract

**Background:**

An increasing number of studies on physicians’ professionalism have been done since the 2002 publication of *Medical Professionalism in the New Millennium: A Physician Charter*. *The Charter* proposed three fundamental principles and ten responsibilities. However, most studies were done in developed countries, and few have been done in China. Additionally, few studies have examined the effect of patient-centered hospital culture (PCHC) on physicians’ professionalism. We aimed to investigate physicians’ medical professionalism in public hospitals in China, and to assess mediating effect of professional attitudes in the relationship of PCHC with professional behaviours.

**Methods:**

Self-administered questionnaires including professional attitudes (20 items) and behaviours (10 items) survey and PCHC scale (22 items) were given to clinical physicians in five public hospitals, China. The mediating effect of professional attitudes in the relationship of PCHC with professional behaviours was tested.

**Result:**

232 valid questionnaires were collected. More than 90% (208) respondents agreed with 15 of 20 specific statements on medical professionalism. As for the responsibility of improving quality of care, 54 (23%) respondents disagreed with reporting of incompetent colleagues and as for the responsibility of maintaining professional competence, 49 (21%) disagreed with recertification. More than 185 (83%) respondents reported that they sometimes, usually, or always showed the four positive behaviours on the questionnaire, and 173 (77%) reported that they never showed the six negative behaviours. Mediating effect analysis revealed that two dimensions of PCHC (i.e. value/institution culture and behaviour/material culture) had a significant positive impact on physicians’ professional behaviour, and professional attitude played a complete mediation role between them, but another dimension of PCHC (i.e. negative evaluation of hospital) directly affected professional behaviour without influencing professional attitude.

**Conclusion:**

Chinese physicians showed positive professional attitudes and behaviours. Different dimensions of PCHC affected physicians’ attitudes and behaviours in different ways.

**Supplementary Information:**

The online version contains supplementary material available at 10.1186/s12910-023-00936-7.

## Introduction

Professionalism is essential in all professions and particularly important in health care system. Since *Medical Professionalism in the New Millennium: A Physician Charter* (*the Charter*) was first published in 2002 [1, 2], it has been endorsed by 109 national and international organizations [3]. The fundamental principles of *the Charter* are the primacy of patient welfare, patient autonomy and social justice, and the ten professional responsibilities are commitment to professional competence, honesty with patients, patient confidentiality, maintaining appropriate relations with patients, improving quality of care, improving access to care, a just distribution of finite resources, scientific knowledge, maintaining trust by managing conflicts of interest and professional responsibilities. *The Accreditation Council on Graduate Medical Education (AGGME)* recommended six general competencies among the medical residents, and one of which was professionalism [4].

In the past 20 years, much attention has been paid to medical professionalism in definition/framing [5–7] and instruments [8], medical education [9, 10], and residency training [11–13]. Meanwhile, an increasing number of empirical studies on clinical physicians’ professionalism have been conducted in USA [14], USA and UK [15], 7 European countries [16], and China [17, 18], and most of them adopted the 3 principles and 10 responsibilities proposed by *the Charter*. It is noteworthy that findings obtained from medical students in educational context may be inappropriate to physicians or residents in real world. For example, a study on residents in Pakistan explored the correlation between empathy level and professionalism, and found absence of correlation [19], although a study among Irish medical students indicated that empathy may act as one of determinants of their attitudes towards medical professionalism [20], which suggesting that more empirical studies are needed to explore the physicians’ actual medical professionalism in practice settings.

Apart from the status quo of physicians’ or residents’ professional attitudes and behaviours, some research has explored the influencing factors of medical professionalism. However, most of these studies focused on personal factors instead of organizational and environmental factors. For example, a qualitative study explored the personal factors affecting medical professionalism in clinical settings, and it extracted five categories (people’s belief in professionalism, personality traits, problems in family, mental or physical health status, and communication skills) from 103 codes [21]. A study on Chinese physicians indicated social support partially mediated the relationship between burnout and professionalism behaviours [22].

As Lesser et al. [23] pointed out that professionalism should be considered in more dynamic and behavioural terms rather static quality and professional behaviours were profoundly influenced by the organizational and environmental context of contemporary medical practice, it was necessary to investigate the effect of organizational factors on physicians’ professionalism. Organizational culture (OC) is defined as a set of beliefs, values, and assumptions that are shared by members of an organization [24], and these underlying values strongly influences organizational members’ behaviours. Numerous empirical literatures have proved that OC is related to outcomes favorable to employees (e.g., job satisfaction, commitment, positive behaviour) and the organization (e.g., performance) [25–27]. Since 1980s, the interest in OC in healthcare field has increased [28]. Evidences in the past 40 years suggest links between organizational culture in hospital (i.e. hospital culture, HC) and adverse events, patient outcomes (such as hospital acquired infections), professional well-being, and hospital performance (such as innovative behaviour) [29–32]. However, few studies have addressed the effect of HC on physicians’ professional attitudes and behaviours. The purpose of this study was to describe the professional attitudes and behaviours of physicians in public hospitals in China and to assess mediating effect of professional attitudes in the relationship of HC with professional behaviours.

## Methods

### Study design and setting

Multistage sampling was used to select the hospitals. Beijing, the capital of China, Wuhan City, the capital of Hubei province and Yingtan City, a prefectural city of Jiangxi province were selected. In each city, one Grade III Level A hospital and one Grade II Level A hospital were selected. In each hospital, two specialties were mainly chosen (internal medicine and general surgery) to ensure comparability. Ultimately, we chose two hospitals in Beijing in eastern China (B1-Grade II Level A, B2-Grade III Level A), one hospital in Hubei Province (W-Grade III Level A, another hospital was given up because the sample size was less than 30), and two hospitals (Y1-Grade II Level A, Y2-Grade III Level A) in Jiangxi Province in central China (Table 1). Except B1 hospital, the other hospitals were teaching hospitals, and none of them was the site of a professionalism seminar.

**Table 1:** Characteristics of respondents in this study (n = 232)

Characteristic	Categories	n/N	%
Gender	Male	110/228	48.2
	Female	118/228	51.8
Age (years)	≤ 25	22/226	9.7
	26~	85/226	37.6
	31~	55/226	24.3
	36~	34/226	15.0
	≥ 41	30/226	13.3
Marital status	Married	149/226	65.9
	Single	76/226	33.6
	Divorced/widowed	1/226	0.4
Working years as a physician(years)	< 4	76/214	35.5
	4~	57/214	26.6
	10~	37/214	17.3
	≥ 15	44/214	20.6
Educational Background ^a^	junior college	15/226	6.6
	Bachelor’s degree	120/226	53.1
	Master’s degree	65/226	28.8
	Doctor’s degree	26/226	11.5
Technical title ^b^	To be appraised	107/224	47.8
	Junior	87/224	38.8
	Middle	22/224	9.8
	Senior	8/224	3.6
employment relationships ^c^	Personnel Establishment/ Staffing of Public Institution	115/222	51.8
	Personnel Agency	44/222	19.8
	Contract	44/222	19.8
	Other	19/222	8.6
Working hours per week	< 44	31/225	13.8
	44~	99/225	44.0
	60~	64/225	28.4
	≥ 80	31/225	13.8
Income per year (RMB)	< 40,000	39/223	17.5
	40,000~	61/223	27.4
	60,000~	34/223	15.2
	80,000~	40/223	17.9
	≥ 100,000	49/223	22.0
Hospital	Y1(Grade II Level A, Jiangxi Province)	34/232	14.7
	Y2(Grade III Level A, Jiangxi Province)	37/232	15.9
	B1(Grade II Level A, Beijing)	65/232	28.0
	B2(Grade III Level A, Beijing)	38/232	16.4
	W1(Grade III Level A, Hubei Province)	58/232	25.0

Notes:

a. In medical education system in China, there are medical majors for both undergraduate and junior colleges. Those with a bachelor’s degree in medical majors can take the practicing physician qualification exam after one year of practice under a physician’s guidance. Individuals with a junior college degree can take the exam after two years of practice with an assistant practicing physician certificate. Those who pass the examinations and obtain the qualifications can apply for registration and practice. Therefore, the respondents could have junior college degrees

b. Physicians can obtain a junior professional title after one year of work following graduation. “To be appraised” refers to that physicians’ professional titles have not been evaluated because they have been working for less than one year

c. Staff with “personnel establishment” is recruited by the administrative department of health and assigned to hospitals. “Personnel agency” refers to the entrusted agency contract signed by the hospital and the personnel agency, the personnel file relationship of the staff is managed by the agency, and the hospital signs the labor contract with the employee. “Contract” means that the hospital directly signs a general labor contract

### Study participants and eligibility criteria

Convenience sampling was used to investigate the physicians. The administrators of the hospital medical service department or human resources office assisted the investigation. They took the researchers to each department to distribute questionnaires and about half an hour later the researchers collected the questionnaire independently. Oral informed consent was obtained from all participants. And this survey was conducted anonymously. Altogether, we distributed the questionnaires to 256 physicians in the five public general hospitals, and 232 valid questionnaires were obtained yielding an overall response rate of 90.6%. Clinical physicians were investigated, and resident physicians and refresher physicians were excluded.

### Study instruments

#### Measurement of medical professional attitudes and behaviours

According to three fundamental principles and ten professional commitments put forward by the *Charter*, based on *the Chinese Medical Physician Declaration* in 2011 and *the Professional Code of Ethics for Chinese Physicians* in 2014 published by Chinese Medical Doctor Association and several instruments in previous studies such as Campbell et al. [14], Roland et al. [15], Lombarts et al. [16] and Chen et al. [17], we developed the professionalism inventory encompassing both professional attitudes and behaviours. The attitude scale consisted of 9 subscales with 20 items: maintaining professional competence (3 items), honesty with patients (3 items), keep patients’ confidentiality (1 item), improving quality of care (5 items), improving access to care (2 items), just distribution of finite resources (1 item), commitment to scientific knowledge (1 item), Maintaining trust by managing conflicts of interest (1 item), and commitment to professional responsibilities (3 items). Professional behaviours included 8 subscales with 10 items: maintaining professional competence (1 item), honesty with patients (1 item), keep patients’ confidentiality (1 item), improving quality of care (1 item), improving access to care (1 item), just distribution of finite resources (2 items), maintaining trust by managing conflicts of interest (2 items), and commitment to professional responsibilities (1 item). The professional attitude items were answered on a 4-point Likert scale (1 = completely disagree, 2 = somewhat disagree, 3 = somewhat agree, and 4 = completely agree). Physicians described the frequency of professional behaviours in the last year on a 4-point Likert scale (1 = never, 2 = sometimes, 3 = usually, and 4 = always). 6 attitude items were reverse scoring. When aggregating the total score, the reversed items were recoded.

### Assessment patient-centered hospital culture (PCHC)

According to the widely-used Schein’s model of OC, OC can be divided into three interrelated levels–basic underlying assumptions, espoused beliefs and values, and artifacts [33]. The basic underlying assumptions were difficult to discern because they were taken for granted beliefs and values unconsciously, while the culture will manifest itself at the level of observable artifacts (the visible products, observed behaviours, and structural elements) and shared espoused beliefs and values. Therefore, two levels of the OC, i.e. artifacts level and the espoused beliefs and values level, were designed to be measured in our study. We developed patient-centered hospital culture (PCHC) inventory draft. Experts in hospital culture research fields and hospital managers were consulted to modify the inventory. The final version of the scale had 22 items. Espoused beliefs and values dimension referred to hospital values and beliefs about patient-centered service (5 items, e.g. “my hospital attaches importance to improve patient satisfaction”), artifacts dimension included three sub-dimensions: (a) material culture was about medical equipment and hospital infrastructure, which was been seen as the visible products (5 items, e.g. “the layout of departments is scientific and reasonable”); (b) behaviour culture was about patient-centered service delivery (6 items, e.g. “activities for quality of care improvement achieved greatly in the hospital”, “there were a series of humanized service systems (e.g. emergency green channel system) in my hospital”); and (c) institution culture referred to patient-centered management system and diagnosis and treatment norms (6 items, e.g. “there are comprehensive medical ethics supervision system in the hospital”). 4-point Likert scale was used (1 = completely disagree, 2 = somewhat disagree, 3 = somewhat agree, and 4 = completely agree). Among the 22 items, 3 items were reverse scoring (e.g. my hospital cannot properly handle patient complaints). The reversed items were recorded in the analysis.

Each item of the respective measures was evaluated by the experienced exports and senior physicians during the design and physicians’ comments and suggestions were collected to modify the measures in pre-investigation till information saturation, which guaranteed content validity.

Exploratory factor analysis about the scale of PCHC showed that KMO value was 0.901, and Bartlett’s test χ^2^ = 2684.953, *P* < 0.001. After excluding one cross-loading item, we get the final scale of PCHC including 21 items. There are three factors with eigenvalues of 1.00 or higher, and 58.997% of the total variation can be explained. The first factor consisted of items about espoused beliefs and values dimension and one sub-dimension of artifacts (i.e. institution culture, such as “my hospital emphasizes that staff should respect and care for patients, and protect the patients’ rights”), the second factor consisted of items about two sub-dimensions of artifacts (i.e. behaviour culture and material culture, such as “the environment and facilities are comfortable, clean and convenient in the hospital”), and the third factor consisted of all the reversed items (such as “staff in different departments seldom communicates with each other”). According to the theoretical assumption and meaning the items, the three factors are renamed: value/institution culture, behavioural/material culture, and negative evaluation of hospital, respectively. The Cronbach’s α of the whole scale and three dimensions are 0.919, 0.911, 0.897 and 0.705, respectively [34].

### Statistical analysis

Data were analyzed using SPSS 24.0 and Amos 24.0. Frequencies and percentages were used to describe categorical variables, and means and standard deviations were used to describe the distributions of continuous variables. The scores differences of different groups were tested by T-test and one-way ANOVA at a significance level of *P* < 0.05. Pearson’s correlation analysis was used to test the relationship between PCHC and professionalism. The mediation analysis was performed by Amos. The indirect, direct, and total effects three dimensions of PCHC on professional behaviour via professional attitude were determined.

## Results

### Participant characteristics

Table 1 summarizes the characteristics of the respondents. Of 256 participants, 232 responded to a questionnaire with an overall response rate of 90.6%. The mean age of the study participants was 32.88 (SD ± 7.14) years which ranges from 21 to 55 years. 110 (48.2%) were male and 118 (51.8%) were female and the majority 271 (66.1%) of the participants were married. In terms of the educational background, almost all 211 (93.4%) participants have a bachelor’s degree or above. About two third 64.5% respondents had above five years of work experience. More than half 117 (52.2%) of participants had junior or above technical title. As for type of affiliation, more than half 115(51.8%) of participants were in personnel establishment (i.e. affiliated with the hospital). As for the hospitals, Y1 and Y2 were from Yingtan city, Jiangxi Province, Central China, B1 and B2 were from Beijing, the capital of China, and W1 was from Wuhan city, the capital of Hubei Province, Central China. Among five hospitals, two were Grade II Level A hospitals and three were Grade III Level A hospitals.

### Physicians’ professional attitudes and behaviours

Table 2 shows physicians’ attitudes toward medical professionalism. More than 90% of respondents agreed (somewhat agree or completely agree) with 15 of 20 statements about 9 professional commitments advocated by *the Chapter*.

**Table 2:** Physicians’ attitudes toward medical professionalism (n, %)

Statements	Completely disagree	Somewhat disagree	Somewhat agree	Completely agree	M ± SD
*A1Maintaining professional competence*					3.48 ± 0.46
A11.Physicians should continually update their knowledge and improve their professional ability.	2(0.9)	7(3.0)	68(29.3)	155(66.8)	3.62 ± 0.59
A12. Physicians should undergo periodic recertification examinations.	18(7.8)	31(13.4)	81(35.1)	101(43.7)	3.15 ± 0.93
A13. Physicians should know medical laws and regulations, such as the *Physician practice law* and *Tort liability law.*	0(0.0)	2(0.9)	71(30.7)	158(68.4)	3.68 ± 0.49
*A2 Honesty with patients*					3.60 ± 0.44
A21. Physicians should inform their patients of the pros and cons of the treatment plan.	0(0.0)	2(0.9)	68(29.3)	162(69.8)	3.69 ± 0.48
A22. Physicians should be realistic, and not mislead patients to unreasonable medical choices.	0(0.0)	6(2.6)	63(27.2)	163(70.3)	3.68 ± 0.52
A23. When a significant medical error occurs, physicians should inform the affected patients and/or their family.	3(1.3)	11(4.8)	98(42.4)	119(51.5)	3.44 ± 0.65
*A3 Protecting patients’ confidentiality*					
A31. Physicians should keep confidential the patient’s medical condition, privacy, and etc.	0(0.0)	1(0.4)	64(27.7)	166(71.9)	3.71 ± 0.46
*A4 Improving quality of care*					3.36 ± 0.54
A41. Physicians should be committed to improving medical quality.	1(0.4)	8(3.4)	68(29.3)	155(66.8)	3.63 ± 0.58
A42. Physicians should participate in peer evaluations of the quality of care provided by colleagues.	3(1.3)	28(12.1)	92(39.7)	109(47.0)	3.32 ± 0.74
A43. Physicians should actively report their adverse medical events.	1(0.4)	11(4.7)	89(38.4)	131(56.5)	3.51 ± 0.61
A44. Physicians should report adverse medical events of other physicians to leaders or relevant departments.	4(1.7)	36(15.5)	90(38.8)	102(44.0)	3.25 ± 0.78
A45. Physicians should report incompetent colleagues to leaders or relevant departments.	4(1.7)	50(21.6)	95(40.9)	83(35.8)	3.11 ± 0.80
*A5 Improving access to care*					3.49 ± 0.54
A51. Physicians should provide essential medical care services regardless of the patient’s ability to pay.	6(2.6)	17(7.4)	85(36.8)	123(53.2)	3.41 ± 0.74
A52. As for the patients with financial difficulties, physicians should choose as economical and effective treatment plan as possible.	0(0.0)	6(2.6)	90(38.8)	136(58.6)	3.56 ± 0.55
*A6 Just distribution of finite resources*					
A61. Physicians should treat patients equally regardless of ethnic, gender, economic status, social status, religion, etc.	1(0.4)	4(1.7)	58(25.0)	169(72.8)	3.70 ± 0.52
*A7 Commitment to scientific knowledge*					
A71. Physicians should popularize health knowledge to the public in an easy to understand way.	0(0.0)	4(1.7)	68(29.3)	160(69.0)	3.67 ± 0.51
*A8 Maintaining trust by managing conflicts of interest*					
A81. Physicians should put patients’ welfare above the physician’s own personal interests.	10(4.3)	26(11.2)	75(32.3)	121(52.2)	3.32 ± 0.84
*A9 Commitment to professional responsibilities*					3.70 ± 0.44
A91. Physicians should follow rules and guidelines for diagnosis, treatment and medication, and provide reasonable medical service.	0(0.0)	4(1.7)	56(24.1)	172(74.1)	3.72 ± 0.49
A92. Physicians should evaluate colleagues’ professional ability and personal qualities fairly and objectively.	0(0.0)	3(1.3)	79(34.1)	150(64.7)	3.63 ± 0.51
A93. Physicians should not slander each other or improperly obstruct patient’s trust in peers.	1(0.4)	2(0.9)	56(24.1)	173(74.6)	3.73 ± 0.49

Table 3 presents physicians’ behaviours in the past one year. More than 185 (83%) respondents reported that they sometimes, usually, or always showed the four positive behaviours on the questionnaire, and 173 (77%) reported that they never showed the six negative behaviours.

**Table 3:** Physicians’ professional behaviours (n, %)

	Never	Sometimes	Usually	Always	M ± SD
In the last year, I …					
*B1 Maintaining professional competence*					
B11. learned and applied new professional knowledge and techniques critically.	17 (7.6)	36 (16.1)	10 (45.3)	69 (30.9)	3.00 ± 0.88
*B2Honesty with patients*					
B21. withheld information that patients or their family should have known about a medical error. (R)	185 (82.6)	29 (12.9)	7 (3.1)	3 (1.3)	1.23 ± 0.57
*B3 Protecting patients’ confidentiality*					
B31. improperly disclosed patient information to irrelevant people. (R)	203 (90.6)	10 (4.5)	6 (2.7)	5 (2.2)	1.17 ± 0.57
*B4 Improving quality of care.*					
B41. participated in medical error reduction activities.	13 (5.8)	55 (24.6)	90 (40.2)	66 (29.5)	2.93 ± 0.88
*B5 Improving access to care*					
B51. provided necessary medical services to patients who are unable to afford it.	39 (17.4)	62 (27.7)	68 (30.4)	55 (24.6)	2.62 ± 1.04
*B6 Just distribution of finite resources*					1.27 ± 0.51
B61. treated the patients differently because of economic status, social status, gender, ethnic, etc. (R)	173 (77.2)	42 (18.8)	6 (2.7)	3 (1.3)	1.28 ± 0.58
B62. provided extra medical services for patients with medical insurance. (R)	184 (82.5)	26 (11.7)	8 (3.6)	5 (2.2)	1.26 ± 0.63
*B8 Maintaining trust by managing conflicts of interest*					1.14 ± 0.39
B81. accepted properties from the patient or their relatives/friends. (R)	197 (87.9)	23 (10.3)	3 (1.3)	1 (0.4)	1.14 ± 0.42
B82. accepted properties, kickbacks, or other unfair benefits from a pharmaceutical company. (R)	197 (88.3)	22 (9.9)	3 (1.3)	1 (0.4)	1.14 ± 0.42
*B9 Commitment to professional responsibilities*					
B91.When needed, I sought help for my colleagues and obtained more reasonable medical plan.	14 (6.3)	32 (14.3)	91 (40.8)	86 (38.6)	3.12 ± 0.88

Note: (R) means reverse scoring

T-test and one-way ANOVA revealed that there are no statistically significant differences between male and female, between single and others, among different ages, working years, educational background, technical title, types of affiliation (personnel establishment and others), working hours per week, even salary per year or different provinces with respect to physicians’ attitudes and behaviours (*P* > 0.05).

The scores for the professional attitudes of physicians in hospitals Y1, Y2, B1, B2, and W1 were 3.42 ± 0.38, 3.53 ± 0.45, 3.66 ± 0.27, 3.43 ± 0.36 and 3.49 ± 0.35, respectively. A one-way ANOVA revealed that there was a statistically significant difference in physicians’ professional attitudes between at least two hospitals (F _(4, 222)_ = 3.755, *P* = 0.006). LSD post-hoc multiple comparisons between hospitals revealed that the physicians’ attitudes in B1 hospital (Grade II Level A) in Beijing were more significantly positive than B2 (Grade III Level A) in Beijing, Y1(Grade II Level A) in Jiangxi Providence, and W1 (Grade III Level A) in Hubei Providence, and there was no significant difference between B1 hospital and Y2 hospital (Grade III Level A). However, there were no significant differences in physicians’ behaviours among different hospitals.

### Relationship between PCHC and professionalism

Table 4 illustrates that each dimension of PCHC is positively related to professional attitudes and behaviours.

**Table 4:** Means, SDs and correlations for PCHC and medical professionalism

	M + SD	PCHC1	PCHC2	PCHC3	attitude	behaviour
PCHC						
PCHC1value/institution	3.46 ± 0.50	1				
PCHC2behaviour/material	2.98 ± 0.61	0.525^***^	1			
PCHC3negative evaluation of hospital	2.80 ± 0.73	0.387^***^	0.474^***^	1		
Medical Professionalism						
attitude	3.52 ± 0.36	0.482^***^	0.368^***^	0.245^***^	1	
behaviour	3.44 ± 0.35	0.198^**^	0.182^**^	0.229^**^	0.330^***^	1

PCHC: patient-centered hospital culture; PCHC1: value/institution culture; PCHC2: behavioural/material culture; PCHC3: negative evaluation of hospital

^*^P < 0.05, ^**^P < 0.01, ^***^P < 0.001

### Mediation effect of professional attitude between PCHC and professional behaviour

To explore the mediating effect of attitude PCHC on behaviour, path analysis was used by Amos. Factor scores of three dimensions of PCHC instead of raw scores were used, and the scores of professional attitude and professional behaviour were standardized. The result shows that χ^2^ = 0.9, df = 3, and therefore χ^2^/3 = 0.3 < 1, and RMSEA < 0.001, which indicated that model fit was good. Table 5 shows the 4 pathways are significant.

**Table 5:** SEM–Estimate, Standard error (SE), Critical ratio (CR), and P-value

pathway			Estimate	SE	CR	P
Attitude	<---	PCHC1	0.477	0.059	8.094	< 0.001
Attitude	<---	PCHC2	0.268	0.058	4.591	< 0.001
Attitude	<---	PCHC3	0.053	0.059	0.889	0.374
behaviour	<---	attitude	0.316	0.077	4.127	<0.001
behaviour	<---	PCHC1	− 0.025	0.075	− 0.335	0.738
behaviour	<---	PCHC2	0.041	0.068	0.597	0.551
behaviour	<---	PCHC3	0.142	0.066	2.149	0.032

Table 6 indicates pathway estimates and 95% confidence intervals (CIs) for the mediation model. As for PCHC1 and PCHC2, the indirect effects’ 95% CIs did not include zero, and the direct effects’ 95% CIs include zero; therefore, there is full mediation between PCHC1/PCHC2 and behaviour. As for PCHC3, the direct effects’ 95% CIs did not include zero, and the indirect effects’ 95% CIs include zero; therefore, there is no mediation between PCHC3 and behaviour.

**Table 6:** Indirect effect of attitude between PCHC and behaviour

Parameter	Estimate	95% CIs		P
		Lower	Upper	
indirect effect 1 (PCHC1–> attitude --> behaviour)	0.151	0.076	0.234	0.023
indirect effect 2 (PCHC2–> attitude --> behaviour)	0.085	0.037	0.153	0.007
indirect effect 3 (PCHC3–> attitude --> behaviour)	0.017	− 0.031	0.057	0.410

**Fig. 1:**
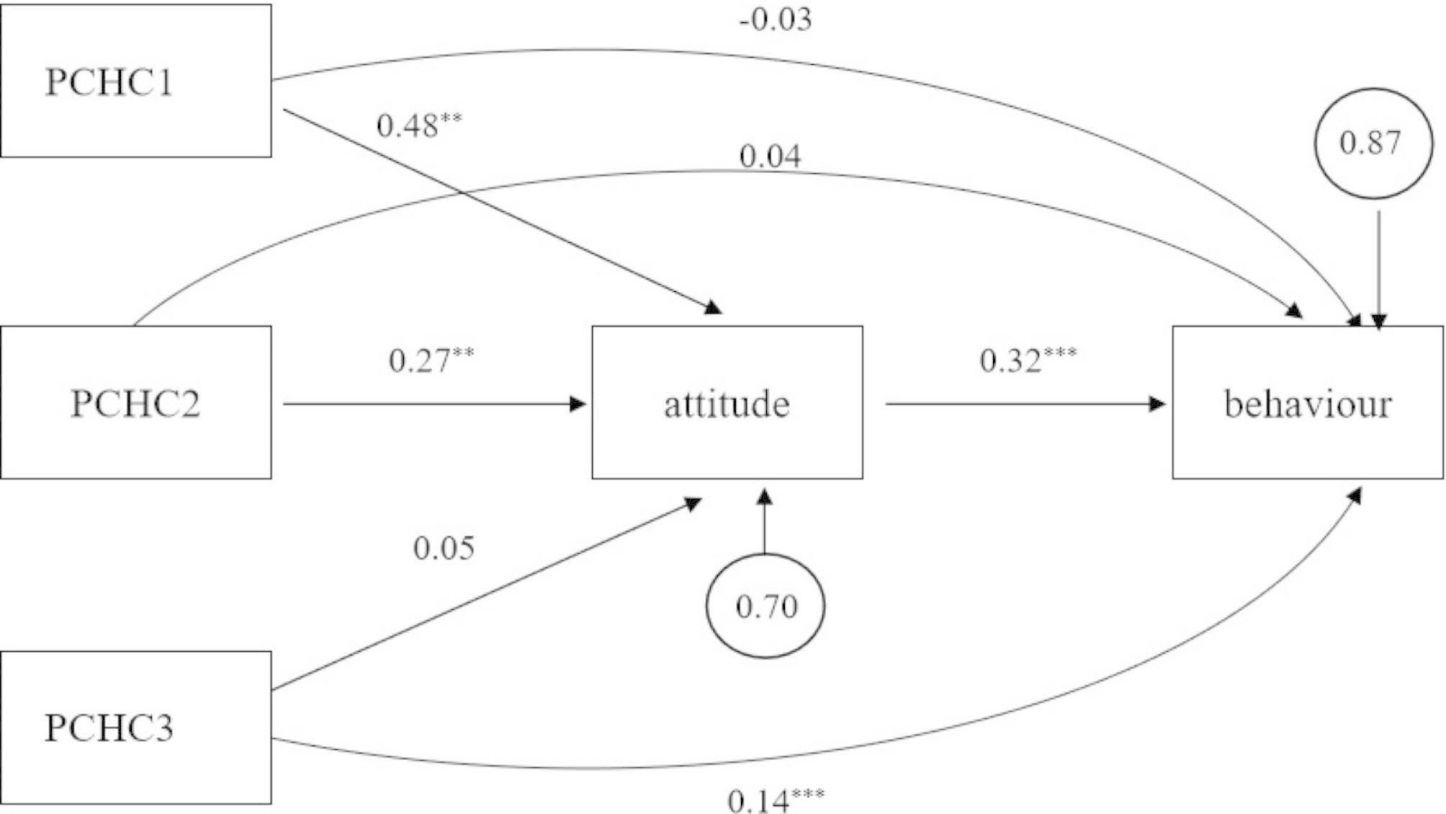
The mediating effect of attitude between PCHC and behaviour ^*^P < 0.05, ^**^P < 0.01, ^***^P < 0.001

That is: PCHC1 and PCHC2 have a significant positive impact on behaviour, and attitude plays a complete mediation role between them; while negative evaluation of hospital directly affects behaviour without influencing attitude (Fig. 1).

## Discussion

We believe that our study is an important attempt to assess Chinese physicians’ medical professionalism and its’ organizational influencing factors quantitatively. Several notable finding emerged in our study.

Firstly, 208 (90%) respondents agreed with 15 of 20 specific statements on medical professionalism, and Chinese physicians demonstrated positive attitudes toward medical professionalism, which is consistent with the results of studies with Chinese samples [17, 18], though the extent to agreement in certain items is the same as or less than their USA and UK counterparts [14, 15]. For example, 78.7% physicians in USA and 82.3% in UK, respectively, strongly agree that physicians should put patients’ welfare above physician’s own personal interests, while 52.2% of Chinese physicians completely agree with it. It may due to profound reasons such as the inadequate Chinese government financial funding (government funding accounts for 9.7% of hospital overall income according to *2020 China Health Statistical Yearbook*), media’s reporting of adverse news negatively affecting perceptions of the doctor-patient relationship [35], lower salary level in China than in developed countries such as USA, and Canada [36]. Meanwhile, Chinese physicians showed more positive behaviours in certain aspect than their USA and UK counterparts [15]. For example, 88.3% Chinese physicians reported that they never received property or kickbacks from pharmaceutical companies (USA 16.7%, UK 26.8%). This may be due to the closer relationship between physicians and pharmaceutical companies in USA and UK [37, 38]. A national survey of 1891 physicians [39] in USA showed that 83.8% of them reported some type of physician-industry relationships (PIRs), and the prevalence of self-reported PIRs in 2009 was lower than in 2004. In China, *Opinions on Carrying out Special Work on Combating Commercial Bribery* was published by general offices of *the Communist Party of China Central Committee and the State Council* in February, 2006 and *Implementation Opinions on Carrying out Special Work on Combating Commercial Bribery in the Field of Pharmaceutical Purchase and Sales* was published by *Ministry of Health and State Administration of Traditional Chinese Medicine* in April, 2006. A series policies and strategies such as zero-markup drug policy [40], *‘Nine Prohibitions’ on Strengthening the Construction of Medical and Health Practice* published in 2013 (explicitly prohibiting the acceptance of kickbacks and patients’ red envelopes) were implemented to regulate physicians’ behaviours. Therefore, there may be less contact between doctors and pharmaceutical companies in China although commercial rebates were somewhat serious about twenty years ago.

Secondly, as for the relationship between professional attitude and behaviour, we found that attitude was one of significant predictor variable of behaviour, which was confirmed in the previous research [16, 17, 41], although there was a gap between attitude and behaviour. For example, 51.5% of physicians “completely agreed” and 42.4% “somewhat agreed” with disclosure of significant medical error, and 82.6% of physicians reported they never withheld information that patients or families should have known about medical error. Gao et al. [42] pointed out that many Chinese doctors would not disclosure of medical errors to the patient unless there are serious long-term consequences because of many reasons. Perhaps the respondents in this study underestimated the medical errors that patients or families “should have known”. For another example, 90.0% of physicians agreed providing essential medical care services regardless of the patient’s ability to pay and 55.0% of physicians usually or always provided it, which may due to the imperfect medical aid system although *Guiding Norms for Emergency Aid Work (Trial)* promulgated by *the National Health and Family Planning Commission* in 2017, it is about emergency services rather than general services. Anyway, the influencing of attitude on behaviour implies that shaping the physicians’ attitudes through medical education and training [43], academic conference, hospital propagandism and advocation are effective ways to promote professional behaviours.

Thirdly, the most striking finding in this study is that PCHC does play an important role in physicians’ professionalism and it affected the physicians’ behaviour in different ways: value/institution culture and behaviour/material culture indirectly affected behaviours through the attitude, while negative evaluation of hospital affected behaviour directly. Although some studies have explored the influencing factors of of medical professionalism, for example participation in decision-making can affect physicians’ behaviours [17], and this paper provides empirical evidence about the effect of PCHC on medical professionalism. Since the professional behaviours in practice settings are profoundly influenced by the organizational and environmental context [23, 44], the physicians’ professionalism is not only a matter of personal attributes, but also an issue of organization and environment [45]. In medical education and residency context, teaching by example was an effective educational method in shaping resident professionalism [46], and in the practice context, the espoused patient-centered values and artifacts (such as institution, behaviour and material) in the hospitals shaped the attitudes and behaviours of members.

In particular, it should be noted that among the three dimensions of PCHC, negative evaluation of hospital, independent of positive evaluation of other two dimensions, can affect physicians’ behaviours directly. Emotions play a vital role in organizing, motivating and sustaining behaviours [47], and negative and positive emotions have a different effect on individual actions and behaviours. Similar to the findings of a consumer–brand relationship (CBR) study [48] that negative emotions towards a brand can translate directly into actions (such as propagating negative word of mouth, avoidance and vengeance) against it, we found negative evaluation influenced physicians’ behaviours. It has significant implications for practice. In addition to focusing on the positive characteristics of PCHC, interventions that aim to promote medical professionalism should consider the negative aspects of PCHC.

## Conclusion

Chinese physicians showed positive professional attitudes and behaviours. Different dimensions of PCHC affected physicians’ attitudes and behaviours in different ways. Two dimensions (i.e. value/institution culture and behaviour/material culture) had a significant positive impact on physicians’ professional behaviour, and professional attitude played a complete mediation role between them, but negative evaluation of hospital directly affected professional behaviour without influencing professional attitude.

### Strengths and limitations

We focus on the clinical physicians’ professional attitudes and behaviours and the effect of PCHC on professional attitudes and behaviours in public hospitals in China. This is one of the early attempts to explore the organizational influencing factors. However, there were some limitations in this study. First, the small and convenience sample may limit the generalizability of the findings to other types of healthcare institutions in China although no significant differences was observed in terms of behaviours among different hospitals. Second, participants were asked to report their behaviours in the Likert scale ranging from “never” to “always” rather than incidence and they completed the attitudes and behaviours items which covered comparable domains at the same time, which might over-valuate the relationship between attitudes and behaviours. Third, the study measured the physicians’ subjective evaluation of PCHC rather than objective assessment, therefore, the focus of the analysis was on the individual level rather on the organization level.

### Electronic supplementary material

Below is the link to the electronic supplementary material.

Additional File 1: Survey of physician’s work status

## Data Availability

The datasets used and/or analyzed during the current study are available from the corresponding author on reasonable request.

## Abbreviations

SD: Standard Deviation
CI: Confidence Interval
SE: Standard Error
CR: Critical Ratio
OC: Organizational Culture
PCHC: Patient-centered Hospital Culture
PCHC1: Value/institution Culture
PCHC2: Behavioural/material Culture
PCHC3: Negative Evaluation of Hospital
