# Supplementary material for: How does patient-centered hospital culture affect clinical physicians’ medical professional attitudes and behaviours in Chinese public hospitals: a cross-sectional study?
Source: BMC Med Ethics. 2023 Aug 2;24:57. doi: 10.1186/s12910-023-00936-7 (PMC10399054; doi:10.1186/s12910-023-00936-7)
Supplement: Supplementary file 1 — Additional File 1: Survey of physician’s work status [file 12910_2023_936_MOESM1_ESM.docx]

**Survey of physician’s work status**

**Dear doctor:**

Thank you for participating in this survey. The survey asks for your views and experiences regarding hospital culture and medial professional attitude and behavior. It is directed to physicians who currently provide direct patient care. The answers you provide will help to improve hospital management and patient-centered hospital culture, and et at.

The questionnaire is designed to take about 8-10 minutes to complete. Your responses are **completely anonymous**. **Neither your name nor any identifying numbers** appear anywhere on this questionnaire.

If you have any questions about the study and specific survey items, please call Dr. Chen Jing, Principal Investigator at 15337148177, 027-83692727, or send e-mail to [chenjing@mails.tjmu.edu.cn](mailto:chenjing@mails.tjmu.edu.cn).

School of Medicine and Health Management,

Tongji Medical College, Huazhong University of Science and Technology

**A. Please indicate how much you agree with each of the following statements**

|  | Completely disagree | | Somewhat disagree | | | Somewhat agree | | Completely agree | |
| --- | --- | --- | --- | --- | --- | --- | --- | --- | --- |
| 1. My hospital pays attention to social responsibility. | | □_1_ | | □_2_ | □_3_ | | □_4_ | |  |
| 2. My hospital actively participates in public welfare activities (such as free medical diagnosis and public assistance). | | □_1_ | | □_2_ | □_3_ | | □_4_ | |  |
| 3. My hospital emphasizes that staff should respect and care for patients, and protect the patients' rights. | | □_1_ | | □_2_ | □_3_ | | □_4_ | |  |
| 4. My hospital attaches importance to improve patient satisfaction. | | □_1_ | | □_2_ | □_3_ | | □_4_ | |  |
| 5. My hospital advocates respecting science and seeking the truth. | | □_1_ | | □_2_ | □_3_ | | □_4_ | |  |
| 6. There are standard operating procedures in the hospital. | | □_1_ | | □_2_ | □_3_ | | □_4_ | |  |
| 7. There are comprehensive medical ethics supervision system in hospital. | | □_1_ | | □_2_ | □_3_ | | □_4_ | |  |
| 8. My hospital encourages physicians to give full consideration to patients' ideas when making a medical decision. | | □_1_ | | □_2_ | □_3_ | | □_4_ | |  |
| 9. There were a series of humanistic service systems (e.g. emergency green channel system) in my hospital. | | □_1_ | | □_2_ | □_3_ | | □_4_ | |  |
| 10. My hospital **cannot** properly handle patient complaints. | | □_1_ | | □_2_ | □_3_ | | □_4_ | |  |
| 11. The hospital is reluctant to provide resources or funds for improving physicians' competency. | | □_1_ | | □_2_ | □_3_ | | □_4_ | |  |
| 12. Staff in different departments seldom communicates with each other. | | □_1_ | | □_2_ | □_3_ | | □_4_ | |  |
| 13. All departments can coordinate smoothly and solve problems for patients efficiently in the hospital. | | □_1_ | | □_2_ | □_3_ | | □_4_ | |  |
| 14. My hospital can properly deal with medical accidents or disputes. | | □_1_ | | □_2_ | □_3_ | | □_4_ | |  |
| 15.Hospital attaches importance to setting examples with high level of medical ethics and conduct. | | □_1_ | | □_2_ | □_3_ | | □_4_ | |  |
| 16. Physicians are willing to follow models. | | □_1_ | | □_2_ | □_3_ | | □_4_ | |  |
| 17. Activities for quality of care improvement achieved greatly in the hospital. | | □_1_ | | □_2_ | □_3_ | | □_4_ | |  |
| 18. The hospital enjoys a high social reputation. | | □_1_ | | □_2_ | □_3_ | | □_4_ | |  |
| 19. The hospital guidance signs are clear. | | □_1_ | | □_2_ | □_3_ | | □_4_ | |  |
| 20. The layout of departments is scientific and reasonable. | | □_1_ | | □_2_ | □_3_ | | □_4_ | |  |
| 21. My hospital has advanced and comprehensive medical equipment. | | □_1_ | | □_2_ | □_3_ | | □_4_ | |  |
| 22. The environment and facilities are comfortable, clean and convenient in the hospital. | | □_1_ | | □_2_ | □_3_ | | □_4_ | |  |

**B1. Please indicate how much you agree with each of the following statements:**

| Physicians should | Completely disagree | | Somewhat disagree | | Somewhat agree | | Completely agree | |  |
| --- | --- | --- | --- | --- | --- | --- | --- | --- | --- |
| A11.continually update their knowledge and improve their professional ability. . . . . . . . . . . . . . . . . . . . . . . . . . . . . . . . . | | □_1_ | | □_2_ | | □_3_ | | □_4_ | |
| A12. undergo periodic recertification examinations. | | □_1_ | | □_2_ | | □_3_ | | □_4_ | |
| A13. know medical laws and regulations, such as the *Physician practice law* and *Tort liability law.* . . . . . . . . . . . . . . . . . . . . | | □_1_ | | □_2_ | | □_3_ | | □_4_ | |
| A21. inform their patients of the pros and cons of the treatment plan. . . . . . . . . . . . . . . . . . . . . . . . . . . . . . . . . . . . . . . . . . . . . | | □_1_ | | □_2_ | | □_3_ | | □_4_ | |
| A22. be realistic, and not mislead patients to unreasonable medical choices. . . . . . . . . . . . . . . . . . . . . . . . . . . . . . . . . . . . | | □_1_ | | □_2_ | | □_3_ | | □_4_ | |
| A23. When a significant medical error occurs, physicians should inform the affected patients and/or their family. . . . . . . . . . . . . . . . . . . . . . . . . . . . . . . . . . . . . . . . . . . | | □_1_ | | □_2_ | | □_3_ | | □_4_ | |
| A31. keep confidential the patient's medical condition, privacy, and etc. . . . . . . . . . . . . . . . . . . . . . . . . . . . . . . . . . . . . . . . . . | | □_1_ | | □_2_ | | □_3_ | | □_4_ | |
| A41. be committed to improving medical quality. | | □_1_ | | □_2_ | | □_3_ | | □_4_ | |
| A42. participate in peer evaluations of the quality of care provided by colleagues. . . . . . . . . . . . . . . . . . . . . . . . . . . . . | | □_1_ | | □_2_ | | □_3_ | | □_4_ | |
| A43. actively report their adverse medical events. | | □_1_ | | □_2_ | | □_3_ | | □_4_ | |
| A44. report adverse medical events of other physicians to leaders or relevant departments . . . . . . . . . . . . . . . . . . . . . . | | □_1_ | | □_2_ | | □_3_ | | □_4_ | |
| A45. report incompetent colleagues to leaders or relevant departments. . . . . . . . . . . . . . . . . . . . .. . .. .. . . . . . . . . . . . . | | □_1_ | | □_2_ | | □_3_ | | □_4_ | |
| A51. provide essential medical care services regardless of the patient’s ability to pay. . . . . . . . . . . . . .. . . . . . . . . . . . . . . | | □_1_ | | □_2_ | | □_3_ | | □_4_ | |
| A52. As for the patients with financial difficulties, physicians should choose as economical and effective treatment plan as possible. . . . . . . . . . . . . . . .. . . . . . . . . . . . . . . . . . . . . . . . | | □_1_ | | □_2_ | | □_3_ | | □_4_ | |
| A61. treat patients equally regardless of ethnic, gender, economic status, social status, religion, etc. | | □_1_ | | □_2_ | | □_3_ | | □_4_ | |
| A71. popularize health knowledge to the public in an easy to understand way. . . . . . . . . . . . . . . . . . . . . . . . . . . . . . . . . . | | □_1_ | | □_2_ | | □_3_ | | □_4_ | |
| A81. put patients' welfare above the physician's own personal interests. . . . . . . . . . . . . .. . . . . . . . . . . . . . . . . . . . . . . . . . | | □_1_ | | □_2_ | | □_3_ | | □_4_ | |
| A91. follow rules and guidelines for diagnosis, treatment and medication, and provide reasonable medical service. . . . . . . . . . . . . .. . . . . . . . . . . . . . . . . . . . . . . . . . . | | □_1_ | | □_2_ | | □_3_ | | □_4_ | |
| A92. evaluate colleagues' professional ability and personal qualities fairly and objectively. . . . . . . . . . . . . . . . . . . . . . | | □_1_ | | □_2_ | | □_3_ | | □_4_ | |
| A93. not slander each other or improperly obstruct patient's trust in peers. . . . . . . . . . . . . . . . .. . . . . . . . . . . . . . . . . . . . | | □_1_ | | □_2_ | | □_3_ | | □_4_ | |

**B2. ln the last year, how often did you …**

|  | **Never** | | **Sometimes** | | | **Usually** | | **Always** | |
| --- | --- | --- | --- | --- | --- | --- | --- | --- | --- |
| B11. learned and applied new professional knowledge and techniques critically. . . . .. . . . . . . . . . . . . . . . . .. . . . . . . . . . . . | | □_1_ | | □_2_ | □_3_ | | □_4_ | |  |
| B21. withheld information that patients or their family should have known about a medical error. . . . .. . . . . . . . . . . . . . . . . . . . . . | | □_1_ | | □_2_ | □_3_ | | □_4_ | |  |
| B31. improperly disclosed patient information to irrelevant people. . . . . .. . . . . . . . . . . . . . . . . . . .. . . . . . . . . . . . . . . . . . . | | □_1_ | | □_2_ | □_3_ | | □_4_ | |  |
| B41. participated in medical error reduction activities. | | □_1_ | | □_2_ | □_3_ | | □_4_ | |  |
| B51. provided necessary medical services to patients who are unable to afford it. . . . . . . . . . . . . . . . .. . . . . . . . . . . . . . . . . . . | | □_1_ | | □_2_ | □_3_ | | □_4_ | |  |
| B61. treated the patients differently because of economic status, social status, gender, ethnic, etc. . . . . . . . . . . . . . . . . . . . . . . . . | | □_1_ | | □_2_ | □_3_ | | □_4_ | |  |
| B62. provided extra medical services for patients with medical insurance. . . . . . . . . . . . . . . . .. . . . . . . . . . . . . . . . . . . . . . . . . . . | | □_1_ | | □_2_ | □_3_ | | □_4_ | |  |
| B81. accepted properties from the patient or their relatives/friends. . . . . . . . . . . . . . . . .. . . . . . . . . . . . . . . . . . . . . . | | □_1_ | | □_2_ | □_3_ | | □_4_ | |  |
| B82. accepted properties, kickbacks, or other unfair benefits from a pharmaceutical company. . . . . . . . .. . . . . . . . . . . . . . . . . . . . . . | | □_1_ | | □_2_ | □_3_ | | □_4_ | |  |
| B91.When needed, I sought help for my colleagues and obtained more reasonable medical plan. . . . . . . . .. . . . . . . . . . . . . . . . . . . | | □_1_ | | □_2_ | □_3_ | | □_4_ | |  |

**C. personal and professional characteristics (please mark one or fill in the blank)**

C1. Please indicate your gender: □_1_ male □_2_ female

C2. your age:______years old; Working years as a physician______ (years)

C3 Marital status: □_1_ Married □_2_ Single □_3_ Divorced/widowed

C4 Please indicate your education background

□_1_ below junior college education

□_2_junior college education □_3_ bachelor degree

□_4_ Master degree □_5_ Doctor degree

C5. Please indicate your technical title

□_1_ To be appraised □_2_ Junior □_3_ Middle □_4_ Senior

C6. Working hours per week

_1_□＜44 _2_□44~ _3_□60~ _4_□≥80

C7. Which of the following options best describes your current employment relationships with the institution? Please select one.

□_1_ Personnel Establishment/Staffing of Public Institution

□_2_ Personnel Agency

□_3_ Contract

□_4_ Other(Please Specify) _______________

C8. Income in last year (RMB)

_1_□<40,000 _2_□40,000~ _3_□60,000~ _4_□80,000 ~

_5_□≥100,000

**Thank you for taking the time to complete this survey!**

Investigator: _________________ date of investigation(yyyy/mm/dd）: ____________

Hospital name: _____________________
